# CXCL5 Promotes Acetaminophen-Induced Hepatotoxicity by Activating Kupffer Cells

**DOI:** 10.3390/ijms241512180

**Published:** 2023-07-29

**Authors:** Kexin Qiu, Yan Pan, Weizhi Huang, Mengyuan Li, Xueqing Yan, Zixiong Zhou, Jing Qi

**Affiliations:** 1 Department of Pathology and Institute of Oncology, The School of Basic Medical Sciences, Fujian Medical University, Fuzhou 350122, China; qiukexin@stu.fjmu.edu.cn (K.Q.); panyan123@stu.fjmu.edu.cn (Y.P.); hwz1741@stu.fjmu.edu.cn (W.H.); limengyuan@stu.fjmu.edu.cn (M.L.); 2 Diagnostic Pathology Center, Fujian Medical University, Fuzhou 350122, China; 3 Department of Biochemistry and Molecular Biology, The School of Basic Medical Sciences, Fujian Medical University, No.1, Xuefu North Road, University Town, Fuzhou 350122, China; yanxueqing@fjmu.edu.cn

**Keywords:** APAP, KCs, inflammation, hepatocytes, CXCL5

## Abstract

Kupffer cells (KCs) play a key part in the pathological process of acetaminophen (APAP)-induced acute liver injury (ALI), the leading cause of acute liver failure in the world. CXC motif chemokine ligand 5 (CXCL5) exerts proinflammatory effects in acute respiratory distress syndrome and arthritis. In the current study, we aim to reveal the effects of CXCL5 on the activation of KCs and the role of CXCL5 in the pathogenesis of APAP-induced hepatotoxicity. The in vivo study, conducted on mice intraperitoneally injected with APAP (300 mg/kg) to establish the ALI model and then treated with Anti-CXCL5 mAb at 30 min and 12 h after the APAP challenge, showed that CXCL5 expression significantly increased in injured livers, and Anti-CXCL5 mAb mitigated the degree of APAP-evoked ALI in mice which was proven through biochemicals and histological examination. Also, neutralization of CXCL5 had no significant effect on APAP metabolism in the liver but exhibited anti-inflammatory effects and ameliorated hepatocellular death in the injured liver. The in vitro data displayed that recombinant mouse CXCL5 treatment promoted APAP-induced cellular toxicity in primary hepatocytes co-cultured with KCs, compared with single-cultured hepatocytes. Consistent with the result, we found that the Anti-CXCL5 mAb gradient decreased LPS-induced expression of inflammatory cytokines in single-cultured KCs. Therefore, CXCL5 could stimulate KCs to produce inflammatory mediators, therefore damaging hepatocytes from APAP toxicity.

## 1. Introduction

Acetaminophen (APAP) is a popular and safe antipyretic and analgesic pharmacon when consumed at a medicinal dose level. However, high doses and extended ingestion of APAP cause acute liver injury (ALI) and are responsible for nearly half of the cases of drug-induced liver injury [1]. APAP overdose leads to centrilobular hepatocyte damage triggered by the depletion of glutathione (GSH) storage and dysregulation of mitochondrial function, which arises from the formation of N-acetyl-p-benzoquinoneimine (NAPQI), the toxic metabolite that is produced from APAP metabolism catalyzed by cytochrome P450 (CYP) family members [2]. The APAP protein adducts induced by NAPQI in mitochondria result in mitochondrial dysfunction, ATP deficiency, DNA fragmentation, and even cellular necrosis.

Excessive APAP induces sterile inflammation in ALI, which constituents the second stage of the pathological process [3]. As is well acknowledged, the necrosis of hepatocytes triggers the release of damage-associated molecular patterns (DAMPs), activating innate immune response and then promoting APAP-induced ALI [4]. N-acetyl cysteine (NAC) is the only FDA-approved antidote against hepatotoxicity following APAP overdose; however, NAC is effective in detoxifying NAPQI if administered within 8 h of APAP ingestion but NAC can cause side effects [5]. Hence, therapy targeting the immune response in ALI has the potential to become an alternative treatment for APAP hepatotoxicity. Specially, resident Kupffer cells (KCs) and infiltrating monocyte-derived macrophages (MoMFs) are the main hepatic macrophages. As ALI damages hepatocytes, liver macrophages are infiltrated, secreting pro-inflammatory cytokines and chemokines, thus facilitating immune cell recruitment and intensifying inflammatory response [6,7]. Therefore, KCs, the main subtype of macrophages in the liver under physiological conditions, play a decisive part in the pathogenesis of APAP-evoked ALI.

Chemokines are mediators secreted to control the infiltration of immune cells into the liver and regulate the activation of nearly all hepatic cell types [8]. CXC chemokine ligand 5 (CXCL5) or epithelial cell-derived neutrophil-activating peptide is a small cytokine belonging to the chemokine family. CXCL5 specially binds to the G-protein-coupled receptor CXCR1/CXCR2, exerting biological effects via either autocrine or non-autocrine cellular pathways. In addition to the role of CXCL5 in anti-infection [9], cancer [10], and obesity [11], CXCL5 is associated with inflammatory diseases such as acute respiratory distress syndrome [9] and arthritis [12] through proinflammatory effects. Therefore, we suspect that CXCL5 may be related to the second stage of APAP-induced ALI.

In this study, functional studies indicated that Anti-CXCL5 mAb significantly improved ALI; Anti-CXCL5 mAb significantly decreased the infiltration and activation of macrophages and reduced the secondary death of liver cells in vivo. Recombinant mouse (rm) CXCL5 treatment promoted APAP-induced cellular toxicity in primary hepatocytes co-cultured with KCs compared with single-cultured hepatocytes; Anti-CXCL5 mAb inhibited KCs in vitro. In summary, this study demonstrated that CXCL5 is critical for KC activation, thereby facilitating the damage of hepatocytes due to ALI. This suggests that targeting CXCL5 could pave the way for developing an alternative approach to the treatment of ALI patients.

## 2. Results

### 2.1. Anti-CXCL5 mAb Treatment Mitigates the Degree of APAP-Induced ALI in Mice

**Figure 1:**
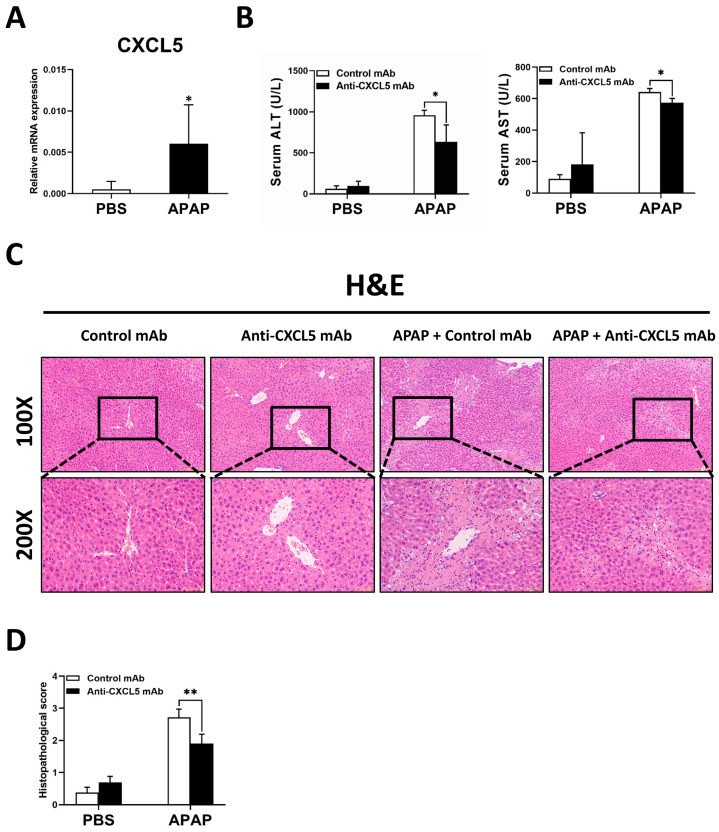
Anti-CXCL5 mAb treatment mitigates the degree of APAP-induced ALI in mice. Mice fasted for 16 h were injected with APAP (300 mg/kg) or the same volume of PBS to induce ALI for comparison. The mice were euthanized at 24 h after the APAP injection. (**A**) The hepatic mRNA expression levels of CXCL5 were detected via qRT-PCR. To verify the function of CXCL5, another batch of mice was injected with APAP (300 mg/kg) or the same volume of PBS to induce ALI, and Anti-CXCL5 mAb (1 μg/mouse) or the same volume of PBS was injected at 30 min and 12 h after the APAP injection. The mice were sacrificed at 24 h after APAP challenge. (**B**) The ALT and AST levels in serum were gauged. (**C**) To show the damage in the liver, H&E staining was performed. (**D**) The necrotic regions in liver histology sections were assessed and scored. The data are expressed as mean ± SD per group (*n* = 5 per group). For the comparisons between the experimental groups, an independent samples *t*-test was used to assess the difference, * *p* < 0.05 and ** *p* < 0.01.

As shown in Figure 1A, CXCL5 expression significantly increased in APAP-evoked ALI mice. To identify the effects of CXCL5 on APAP overdose, mice were challenged with APAP or the same volume of PBS to induce ALI, and Anti-CXCL5 mAb or the same volume of PBS was treated at 30 min and 12 h after APAP injection. It is shown that the level of acute liver damage in Anti-CXCL5 mAb treated mice significantly decreased (Figure 1B–D). Anti-CXCL5 mAb treatment significantly decreased the serum levels of serum alanine transaminase (ALT) and aspartate transaminase (AST) (Figure 1B). This was also proven by the hematoxylin/eosin (H&E) staining and the histopathological score (Figure 1C,D).

### 2.2. Anti-CXCL5 mAb Treatment Has No Significant Effect on Liver Metabolism in ALI Induced by APAP

**Figure 2:**
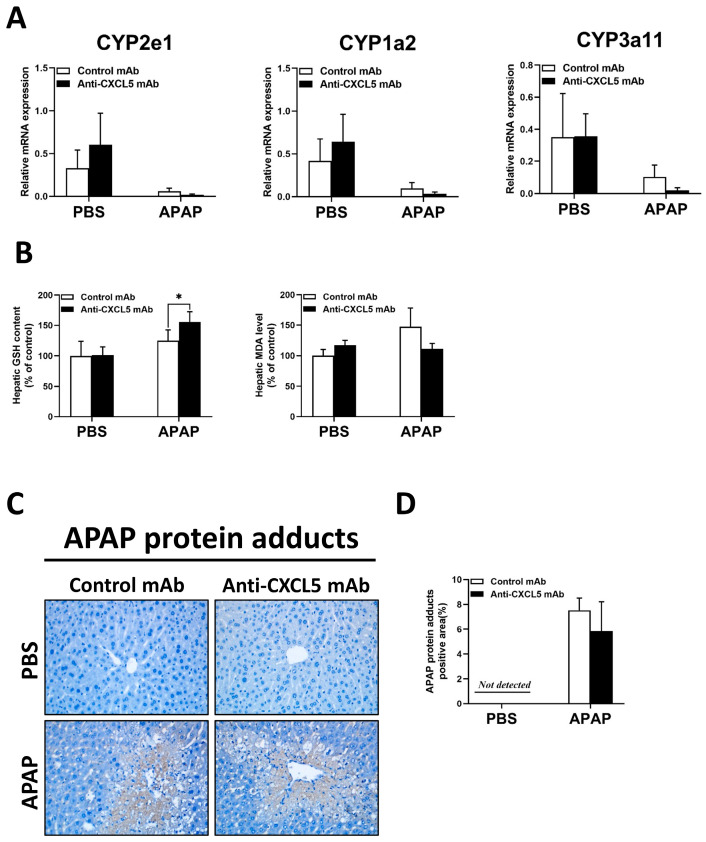
Anti-CXCL5 mAb treatment has no significant effect on liver metabolism in ALI induced by APAP. (**A**) The hepatic mRNA expression levels of CYP enzymes were measured via qRT-PCR. (**B**) The hepatic GSH contents and MDA levels were identified to reveal the oxidative stress conditions. (**C**) To evaluate the distribution of APAP protein adducts, the APAP protein adducts positive areas in liver sections were assessed via IHC staining with an anti-APAP antibody. (**D**) The percentage of APAP protein adducts positive areas were measured. Values are presented as mean ± SD per group (*n* = 5 per group). An independent samples *t*-test was used to assess the difference for the comparisons between the experimental groups, * *p* < 0.05. Original magnification: ×400 (IHC).

It is reported that the CYP family plays an important role in liver metabolism [13]. However, as shown in Figure 2A, there was no significant change in the expression level of the CYP family after Anti-CXCL5 mAb treatment. With the increase in GSH, the antioxidant capacity of the liver enhanced after Anti-CXCL5 mAb treatment, but there was no significant difference in malondialdehyde (MDA) level (Figure 2B).

Formation of APAP protein adducts is central to APAP-induced liver injury and their removal through autophagy is an essential adaptive response after an acute overdose [14]. There was little difference in the APAP protein adducts positive area after Anti-CXCL5 mAb treatment (Figure 2C,D). However, Anti-CXCL5 mAb treatment decreased the expression of nuclear factor erythroid 2-related factor 2 (Nrf2)-target genes including NADPH: quinone oxidoreductase 1 (NQO-1), heme oxygenase-1 (HO-1), glutamate cysteine ligase (GCL) C, and GCLM (Supplementary Figure S1A) in injured livers of mice, which is contrary to our expectations.

### 2.3. Anti-CXCL5 mAb Exhibits Anti-Inflammatory Effects in APAP-Injected Mice

**Figure 3:**
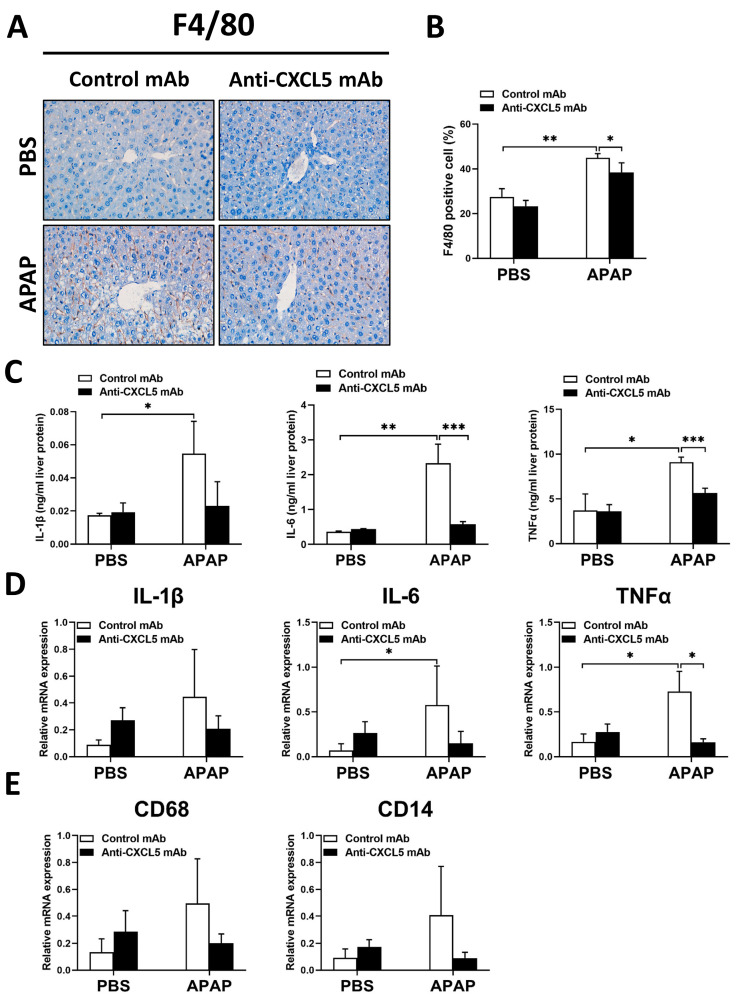
Anti-CXCL5 mAb exhibits anti-inflammatory effects through macrophages in mice with APAP injection. (**A**) The F4/80 positive macrophages were assessed via IHC staining to evaluate hepatic macrophage infiltration. (**B**) The quantification of the percentage of F4/80 positive cells was revealed. (**C**) The hepatic protein levels of IL-1β, IL-6, and TNFα were determined via Elisa. (**D**) The hepatic mRNA expression levels of IL-1β, IL-6, and TNFα were measured via qRT-PCR. (**E**) The gene expression levels of mononuclear macrophage-related chemokines such as CD68 and CD14 were detected using qRT-PCR. The data are presented as mean ± SD per group (*n* = 5 per group). For the comparisons between the experimental groups, an independent samples *t*-test was used to assess the difference, * *p* < 0.05, ** *p* < 0.01, and *** *p* < 0.001. Original magnification: ×400 (IHC).

Since Anti-CXCL5 mAb treatment has no significant effect on liver metabolism in ALI induced by APAP, we next examined whether treating Anti-CXCL5 mAb affects the hepatic inflammation process. As shown in Figure 3A,B, the infiltration of F4/80 positive macrophages elevated in APAP-injected mice, while it reduced in Anti-CXCL5 mAb-treated mice. Compared to the control group, APAP increased the hepatic protein levels or the hepatic mRNA expression levels of interleukin 1 beta (IL-1β), interleukin 6 (IL-6), and tumor necrosis factor-alpha (TNFα), whereas Anti-CXCL5 mAb treatment, to a certain extent, diminished the production of IL-6, TNFα, and IL-1β induced by APAP injection (Figure 3C,D). Furthermore, Anti-CXCL5 mAb treatment showed a tendency to reduce the gene expression levels of mononuclear macrophage-related chemokines such as CD68 and CD14 in APAP-injured livers, but the difference was not statistically significant (Figure 3E).

**Figure 4:**
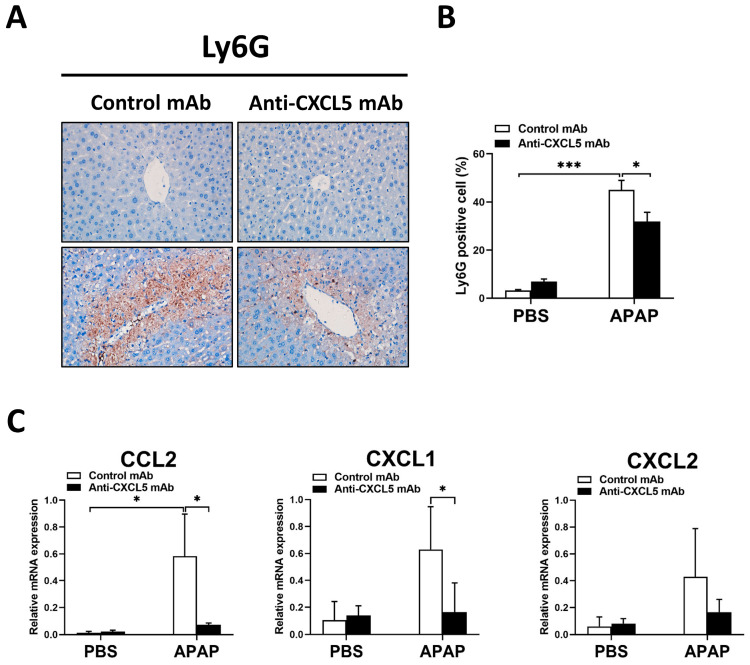
Anti-CXCL5 mAb participates in the anti-inflammatory process in connection with neutrophils in APAP-injected mice. (**A**) The Ly6G positive neutrophils in histological sections of the liver were detected with IHC to reveal hepatic neutrophil infiltration. (**B**) The quantification of the percentage of Ly6G positive cells was shown. (**C**) The gene expression levels of CCL2, CXCL1, and CXCL2 which are neutrophil-related chemokines were evaluated via qRT-PCR. The data are expressed as mean ± SD per group (*n* = 5 per group). For the comparisons between the experimental groups, an independent samples *t*-test was used to assess the difference, * *p* < 0.05 and *** *p* < 0.001. Original magnification: ×400 (IHC).

Similar to Figure 3 which shows the involvement of macrophages, Figure 4 shows the involvement of neutrophils. As shown in Figure 4A,B, the infiltration of Ly6G positive neutrophils increased in injured livers, while decreased in Anti-CXCL5 mAb-treated mice. Additionally, Anti-CXCL5 mAb treatment reduced the gene expression levels of CCL2 and CXCL1 but not CXCL2 which are neutrophil-related chemokines (Figure 4C).

To sum up, these results suggest that Anti-CXCL5 mAb exhibits anti-inflammatory effects by acting on macrophages and neutrophils in APAP-injected mice.

### 2.4. Treatment with Anti-CXCL5 mAb Ameliorates Hepatocellular Death of APAP-Evoked ALI in Mice

**Figure 5:**
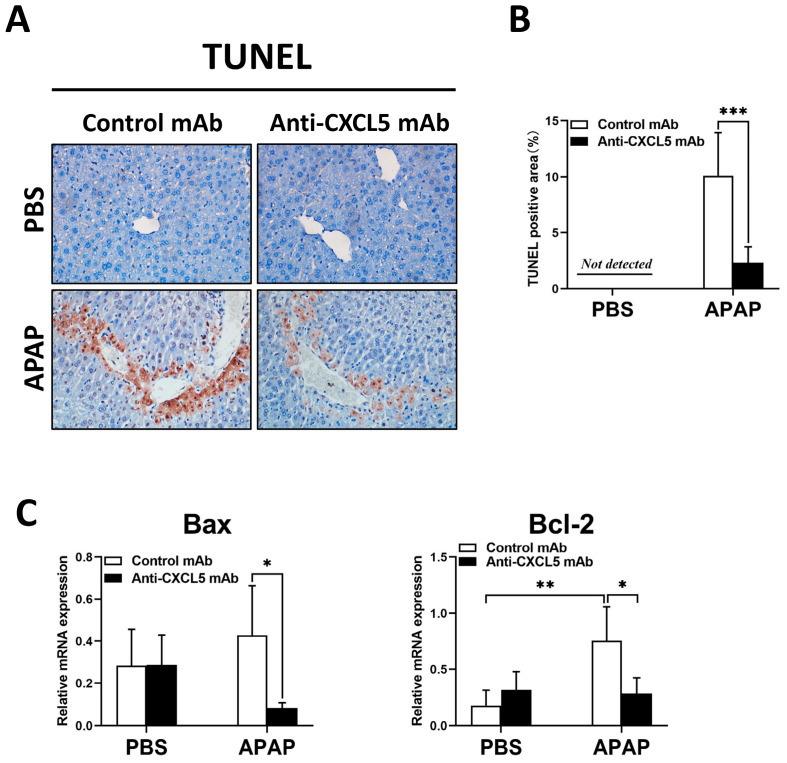
Treatment with Anti-CXCL5 mAb ameliorates hepatocellular death of APAP-evoked ALI in mice. (**A**) TUNEL staining was performed to observe the death of hepatocytes. (**B**) The quantification of TUNEL-positive areas was revealed. (**C**) The hepatic mRNA expression levels of Bax and Bcl-2 were determined via qRT-PCR. Values are presented as mean ± SD per group (*n* = 5 per group). An independent samples *t*-test was used to assess the difference for the comparisons between the experimental groups, * *p* < 0.05, ** *p* < 0.01, and *** *p* < 0.001. Original magnification: ×400 (TUNEL).

As shown in Figure 5A,B, the TUNEL positive area was enlarged in APAP-injected mice compared to the control group, while Anti-CXCL5 mAb treatment reduced the difference. In addition, Anti-CXCL5 mAb treatment reduced the hepatic mRNA expression levels of Bax and Bcl-2, which are the apoptotic genes showing the severity of hepatocellular death (Figure 5C).

### 2.5. rmCXCL5 Stimulates KCs to Produce Inflammatory Mediators In Vitro

**Figure 6:**
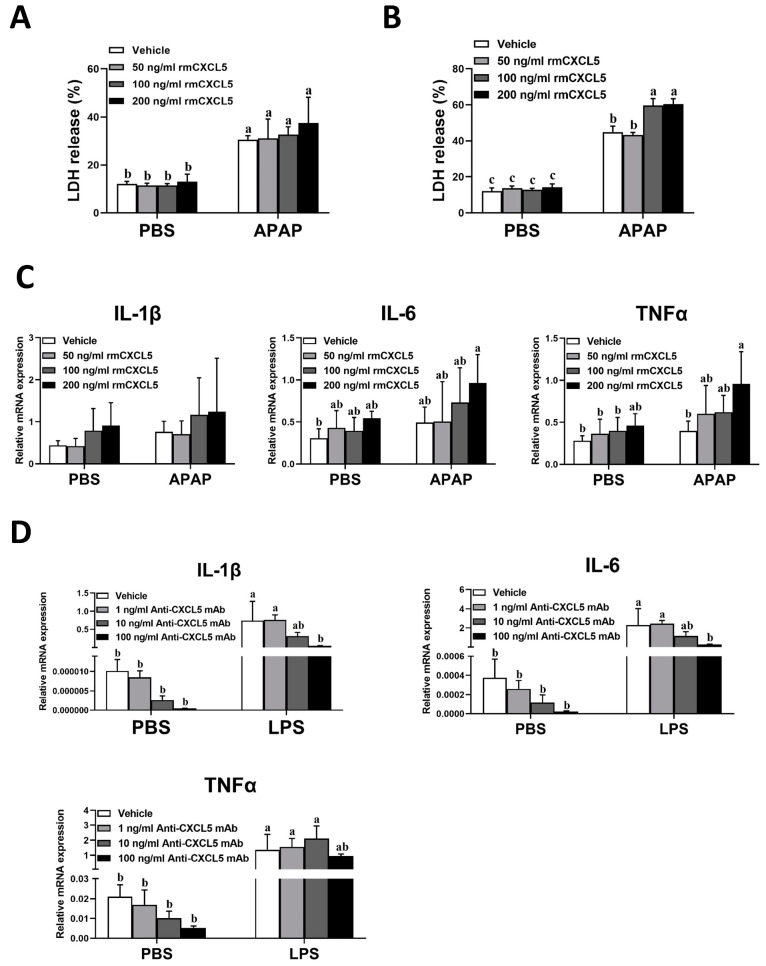
rmCXCL5 stimulates KCs to produce inflammatory mediators in vitro. (**A**) Primary hepatocytes were single-cultured in vitro. Hepatocytes were challenged with APAP (10 mM) to establish an ALI model in vitro, and rmCXCL5 gradient (0, 50, 100, and 200 ng/mL) was administered. The cell samples were gathered at 24 h after APAP stimulation. LDH assay was performed to reveal cytotoxicity. (**B**,**C**) Primary hepatocytes were co-cultured with KCs in vitro. Hepatocytes were challenged with APAP (10 mM) and treated with rmCXCL5 gradient (0, 50, 100, and 200 ng/mL). The cell samples were gathered at 24 h after APAP treatment. (**B**) The cytotoxicity was detected using LDH assay. (**C**) The mRNA expression levels of IL-1β, IL-6, and TNFα were measured via qRT-PCR. (**D**) Macrophages were single-cultured in vitro. Macrophages were stimulated with LPS to simulate liver injury, and gradient Anti-CXCL5 mAb (0, 1, 10, and 100 ng/mL) was administered. The cells were gathered at 12 h after LPS stimulation. The mRNA expression levels of the above cytokines were determined. The data are presented as mean ± SD per group (*n* = 5 per group). The one-way analysis of variance (ANOVA) was performed to assess the difference among the mean values of more than two independent groups. Statistical differences among multiple groups at *p* < 0.05 are represented by different letters.

To further explore the molecular mechanism of CXCL5 in APAP-induced ALI in mice, hepatocytes were challenged with APAP to induce an in vitro ALI model, and rmCXCL5 gradient was administered. Interestingly, the release of LDH had no significant difference between groups of rmCXCL5 gradient treatment in single-cultured hepatocytes, whereas the release of LDH increased significantly between groups of rmCXCL5 gradient treatment in hepatocytes co-cultured with KCs (Figure 6A,B). Moreover, the mRNA expression levels of TNFα, but not IL-1β and IL-6, elevated dose-dependently in the stimulation of rmCXCL5 gradient in co-cultured hepatocytes with APAP treatment (Figure 6C). To simulate the environment of liver injury, KCs were stimulated with LPS and treated with the Anti-CXCL5 mAb gradient. The mRNA expression levels of IL-1β, IL-6, and TNF-α decreased in the treatment with the Anti-CXCL5 mAb gradient (Figure 6D).

In summary, similar to LPS, rmCXCL5 stimulates KCs to produce inflammatory mediators, which aggravates the severity of APAP-induced ALI.

## 3. Discussion

Chemokines and receptors could be potential drug targets for the therapy of liver diseases [8]. In this study, we investigated the pro-inflammatory activity of CXCL5 binding with CXCR1/CXCR2 [12,15]. CXCR1 and CXCR2 are mainly expressed in macrophages and neutrophils [16]. A previous study reported that, as the main inflammatory chemokine pathway, CXCL5/CXCR2 increased inflammation-related neural repair with CD68^+^ cell activation in an optic nerve injury mouse model [17]. Similarly, CXCL5 activated neutrophils to improve the metastatic ability of gastric cancer cells [18]. Consistent with these findings, our study innovatively revealed that CXCL5 could stimulate KCs to produce inflammatory mediators, therefore facilitating the damage of hepatocytes via APAP toxicity in mice, using a co-culture system in vitro. Thus, targeting CXCL5 could provide new insights for developing an alternative approach to the treatment of ALI patients.

Overdose of APAP to induce ALI is a well-established model, which triggers “two hits” during the pathological process [19]. Hepatocyte necrosis is induced directly by the toxic metabolite NAPQI; then, the severity of the hepatic injury and cell death is affected by the immune response induced by the initial damage. Since immune cells have two opposite reactions, pro-inflammatory and anti-inflammatory, the role of immune response in APAP-induced ALI is highly controversial [4,20]. Studies reported that activated macrophages could engulf necrotic cell debris to promote liver regeneration in ALI [6,21]. The contrasting effects of macrophages are determined by their polarization between pro-inflammatory M1 and anti-inflammatory M2 phenotypes responding to various factors [22]. M1 macrophages exacerbate the severity of ALI by increasing the production of pro-inflammatory cytokines and chemokines, and M2 macrophages function as phagocytes to remove cell debris and secrete anti-inflammatory cytokines [23]. Therefore, the regulation of macrophages could provide a new opportunity for the treatment of ALI. In the current study, we showed that Anti-CXCL5 mAb exerts anti-inflammatory properties, observed by the inhibited activation of KCs and the production of inflammatory mediators. This alleviates the hepatocyte damage caused by APAP both in vitro and in vivo.

Nrf2, a nuclear transcription factor, and transcriptional activating antioxidant enzymes including HO-1, NQO-1, and GCL catalyze the initial and rate-limiting step in GSH synthesis [24]. These Nrf2-activated enzymes serve as a cell defense system to detoxify NAPQI [25,26], and Nrf2 activators have been reported to be protective against APAP hepatotoxicity [27,28]. In addition to its role in redox homeostasis, Nrf2 is essential for the inhibition of inflammation by interacting directly or indirectly with important components such as the Toll-like receptors and the type-I interferon response [29]. In the current study, the data showed that Anti-CXCL5 mAb downregulates the gene expression of Nrf2-target genes in injured livers of mice (Supplementary Figure S1), while Anti-CXCL5 mAb treatment enhanced the antioxidant capacity with the increase in GSH (Figure 2B), and reduced infiltration and activation of macrophages and neutrophils (Figure 3 and Figure 4) in livers of mice injected with APAP. These results suggest a minor function of Nrf2 and Nrf2-target genes in the regulation of oxidative stress and inflammation by CXCL5 in ALI mice.

Moreover, the infiltration of neutrophils in ALI mice decreases after Anti-CXCL5 mAb treatment (Figure 4). Neutrophils are attracted to the damaged region of the liver of ALI mice, and damping neutrophil migration into the liver attenuates APAP hepatotoxicity [30]. In this study, the results demonstrated that CXCL5 could affect KC-mediated inflammatory responses in the ALI model. However, further studies are encouraged to appraise the impacts of CXCL5 on neutrophils and other immune cells. Additionally, in the early phase of APAP overdose, NAC preserves hepatocytes by generating GSH. Unfortunately, its therapeutic window is finite, because it has no significant influence in the late inflammatory phase of ALI. Drugs targeting CXCL5 can be used in combination with the NAC for ALI therapy. Therefore, further research should evaluate the impacts of the combined treatment.

## 4. Materials and Methods

### 4.1. Animals

We used seven-week-old male mice (C57BL/6J, Wushi Animal, Fuzhou, China) in this study. All mice were fed in laboratory in vivo facilities with autoclaved food and water, within a 12 h day–night cycle and temperature/humidity-regulated environment (50 ± 5% humidity, 24 ± 2 °C). All experiments were approved by the Experimental Animal Ethical Committee of the Fujian Medical University (FJMU IACUC 2021-J-0555).

### 4.2. Animal Experiments

To investigate the role of CXCL5 in APAP-evoked ALI, twenty mice were randomly divided into four groups [PBS + vehicle, *n* = 5; PBS + Anti-CXCL5 mAb, *n* = 5; APAP + vehicle, *n* = 5; and APAP + Anti-CXCL5 mAb, *n* = 5]. After 16 h of starvation, to induce ALI in mice, APAP (300 mg/kg, Med Chem Express LLC) was intraperitoneally (i.p.) injected, while an equal volume of phosphate-buffered saline (PBS, Life-iLab, Shanghai, China) was administered to the control groups. Anti-CXCL5 mAb (1 μg/mouse, GlpBio) or the same volume of control mAb was i.p. injected at 30 min and 12 h after the APAP challenge. The mice were anesthetized at 24 h after the APAP injection, and we collected the blood and liver samples for further analysis.

### 4.3. Histopathologic Examination

Liver tissues were fixated within a 4% paraformaldehyde solution, embedded in paraffin, and cut to produce 5 μm thick slices. Deparaffinized and rehydrated sections underwent staining with H&E. The H-SCORE was employed to semi-quantify H&E staining for each tissue sample. The score was based on the following guidelines: Grade 0 (no histopathologic alterations), Grade 1 (presence of degenerative cells and rare cases of necrosis), Grade 2 (slight lesion to the entire centrilobular area), Grade 3 (moderate lesion), and Grade 4 (severe lesion).

To detect apoptotic cells in the liver tissues, TUNEL staining (In Situ Cell Death Detection Kit, Roche, Switzerland) was implemented according to the manufacturer’s operating procedures. TUNEL assay uses terminal deoxynucleotidyl transferase (TdT) and shows the positive cells with damaged DNA. Image J (V1.8.0.112) software was used to calculate the percentage of TUNEL positive area in each section.

### 4.4. Biochemical Measurements

ALT and AST activities were detected through the appropriate analytical assay kits (Nanjing Bioengineering Institute, Nanjing, China). Lipid Peroxidation MDA Assay Kit and a GSH and GSSG quantification kit were used to measure hepatic contents of MDA and GSH, respectively. MDA Assay Kit and GSH and GSSG quantification kit were bought from Beyotime, Shanghai, China.

### 4.5. Immunohistochemistry (IHC) Staining

Paraffin blocks were sectioned, de-waxed with xylene, and rehydrated with graded series of alcohol. To observe the hepatic contents of APAP protein adducts and infiltrations of macrophages and neutrophils, liver slides were incubated for 1 h at 37 °C with antibodies of anti-APAP (#0016-0104, Bio-rad; and diluent ratio, 1:250), anti-F4/80 (#sc-377009, Santa Cruz; and diluent ratio, 1:200), and Ly6G (#sc-53515, Santa Cruz; and diluent ratio, 1:200), respectively. Then, sections were treated at 37 °C for 30 min with the secondary antibody (MXB Biotechnologies, Fuzhou, China) and eventually counterstained with hematoxylin. The percentage of positive cells was quantified using the Image J (V1.8.0.112) software.

### 4.6. Quantitative Real-Time Polymerase Chain Reaction (qRT-PCR)

The RNA extraction procedures are as follows. First, Trizol (500 μL) was added to the microcentrifuge tubes with the liver tissues or cell samples. After homogenization, chloroform (100 μL) was added to the tubes. After oscillation, the samples were incubated for 5 min in ice and centrifuged at 12,000 rpm, 4 °C for 10 min. The supernatants (upper layer) were transferred to clean microcentrifuge tubes. After that, isopropanol was added, and then the samples were mixed upside down and incubated for 1 min in ice. After centrifuging, the supernatants were removed. Soon, the pellet was washed with 10 mL of 75% ethanol. The supernatants were completely removed and air-dried for 15 min. Finally, the pallets were dissolved with 20 μL of DEPC water, and then the concentration was measured.

Total RNA (1 μg) was prepared for cDNA synthesis using a reverse transcription system (Fujian Herui Biotechnology Co., Ltd., Fuzhou, China). Gene expression levels were measured via qRT-PCR using an SYBR Green reagent (Fujian Herui Biotechnology Co., Ltd., Fuzhou, China). The relative gene expression levels were normalized by the comparative Ct value of glyceraldehyde-3-phosphate dehydrogenase gene expression. The sequences of primers are included in Table 1.

### 4.7. Cell Cytotoxicity Assays (LDH)

We used an LDH cytotoxicity detection kit (Nanjing JianCheng) to determine LDH, which was released from the damaged hepatocytes. The absorbance at a wavelength of 490 nm was detected via the Emax Precision Microplate Reader (ThermoFisher Scientific Oy Ratastie, Vantaa, Finland).

### 4.8. Enzyme-Linked Immunosorbent Assay (ELISA)

We measured the levels of IL-1β, IL-6, and TNFα using ELISA kits (Boster Biological Technology Co., Ltd., Wuhan, China), which evaluates the severity of the inflammation.

### 4.9. Cell Isolation and Culture

Single-cultured hepatocytes: Primary hepatocytes were isolated from C57BL/6J male mice (eight-week-old). Liver tissue was infused with type I collagenase (1 mL/min), and hepatocytes were isolated through centrifugation (50 g and for 3 min). The precipitated hepatocytes were resuspended and filtered using DMEM (Sigma-Aldrich, St. Louis, MO, USA). Primary murine hepatocytes (5.0 × 10^4^ cells/well) were inoculated into 24-well plates, and cultured in DMEM supplemented with 10% fetal bovine serum (FBS, Sigma-Aldrich), 100 IU/mL penicillin (Life-iLab, Shanghai, China), and 100 μg/mL streptomycin (Life-iLab, Shanghai, China). After cell attachment, 10 mM APAP was added to each of the 12 wells, and the PBS was added to other 12 wells. Half an hour later, the 12 wells in the APAP group were divided into 4 groups, and rmCXCL5 (0, 50, 100, and 200 ng/mL) was added. The PBS group was treated in the same way. Twenty-four hours after treatment, cells and cell supernatant were collected for further experiments.

Co-cultured hepatocytes: Hepatocytes were isolated via centrifugation (50 g and for 3 min), and non-parenchymal cell (NPCs) were purified using Percoll gradient centrifugation. KCs were then further extracted from NPCs using magnetically activated cell sorting with F4/80 antigen-conjugated microbead antibodies. In a co-culture system, primary hepatocytes (5.0 × 10^4^ cells/well) were inoculated into 24-well type I collagen-coated plates, and KCs (1.25 × 10^4^ cells/well) were inoculated in a 4:1 ratio (hepatocyte/KC) into cell culture inserts with 0.4 μm pore polycarbonate membrane (Sigma-Aldrich, Missouri, USA). These cells were cultured in DMEM with 10% FBS and antibiotic–antifungal solution. After cell attachment, 10 mM APAP was added to each of the 12 wells. In the other 12 wells PBS was added. Half an hour later, the 12 wells in the APAP group were divided into 4 groups, and rmCXCL5 (0, 50, 100, and 200 ng/mL) was added. The PBS group was treated in the same way. Twenty-four hours later, cell samples and cell supernatant were collected for further analysis.

### 4.10. Statistical Analysis

Data were shown as the mean ± standard deviation (SD) and the statistical significance was established at *p* < 0.05. All data were analyzed using GraphPad Prism 9. The normality of distribution was analyzed using the Kolmogorov–Smirnov test. Since the data passed tests for normality and homogeneity of variance, parametric tests were used. For the comparisons between the two groups, an independent samples *t*-test was used to assess the difference, * *p* < 0.05, ** *p* < 0.01, and *** *p* < 0.001. The one-way analysis of variance (ANOVA) was performed to assess the difference among the mean values of more than two independent groups, and the significant differences between the groups were indicated with different letters.

## 5. Conclusions

In general, CXCL5 could stimulate KCs to produce inflammatory mediators, therefore facilitating the damage of hepatocytes via APAP toxicity. Furthermore, Anti-CXCL5 mAb substantially reduces ALI in mice by restraining the activation of KCs and KC-mediated inflammatory responses. In summary, our findings discovered a formerly unidentified role of CXCL5 in APAP-induced ALI, which suggests that targeting CXCL5 can be used to treat ALI.

## Figures and Tables

**Table 1:** The primer sequences of genes.

Gene	Gene Accession Number	Forward (5′-3′)	Reverse (5′-3′)
CYP2e1	NM_021282	AAGCGCTTCGGGCCAG	TAGCCATGCAGGACCACGA
CYP1a2	NM_009993.3	GGTCAGAAAGCCGTGGTTG	GACATGGCCTAACGTGCAG
CYP3a11	NM_007818.3	CGCCTCTCCTTGCTGTCACA	CTTTGCCTTCTGCCTCAAGT
TNFα	NM_013693.3	GTCTACTCCCAGGTTTCTCTTCAAGG	GCAAATCGGCTGACGGTGTG
IL-1β	NM_008361.4	CTCGCAGCAGCACATCAACA	CCACGGGAAAGACACAGGTA
IL-6	NM_001314054.1	CAACGATGATGCACTTGCAGA	CTCCAGGTAGCTATGGTACTCCAGA
CXCL1	NM_008176.3	TGCACCCAAACCGAAGTC	GTCAGAAGCCAGCGTTCACC
CXCL2	NM_009140.2	GCCAAGGGTTGACTTCAAGAACA	AGGCTCCTCCTTTCCAGGTCA
CCL2	NM_011333.3	AGCAGCAGGTGTCCCAAAGA	GTGCTGAAGACCTTAGGGCAGA
CD68	NM_009853	ACCGCCATGTAGTCCAGGTA	ATCCCCACCTGTCTCTCTCA
CD14	NM_009841	GGCCGCGCGGATTCCTAGTC	ATCGGGTCCGGTGGCTTCCA
Bax	NM_007527	AAGCGCTTCGGGCCAG	TAGCCATGCAGGACCACGA
Bcl2	NM_177410	GGTCAGAAAGCCGTGGTTG	GACATGGCCTAACGTGCAG
NQO-1	NM_008706	CAGCCAATCAGCGTTCGGTA	CTTCATGGCGTAGTTGAATGATGTC
HO-1	NM_010442	TGCAGGTGATGCTGACAGAGG	GGGATGAGCTAGTGCTGATCTGG
GCLC	NM_010295	AATGACTGTTGCCAGGTGGATG	GGTTGCACTTCCAAATGAGGCTA
GCLM	NM_008129	AGTTGGAGCAGCTGTATCAGTGG	TTTAGCAAAGGCAGTCAAATCTGG
GAPDH	NM_001411840.1	ACGGCAAATTCAACGGCACAG	GAAGACTCCACGACATACTCAGCAC

## Data Availability

All data are included in this manuscript.

## Supplementary Materials

The following supporting information can be downloaded at: https://www.mdpi.com/article/10.3390/ijms241512180/s1.
