# Supplementary material for: CXCL5 Promotes Acetaminophen-Induced Hepatotoxicity by Activating Kupffer Cells
Source: Int J Mol Sci. 2023 Jul 29;24(15):12180. doi: 10.3390/ijms241512180 (PMC10419303; doi:10.3390/ijms241512180)
Supplement: Supplementary file 1 [file ijms-24-12180-s001.zip › ijms-2499537-supplementary.pdf]

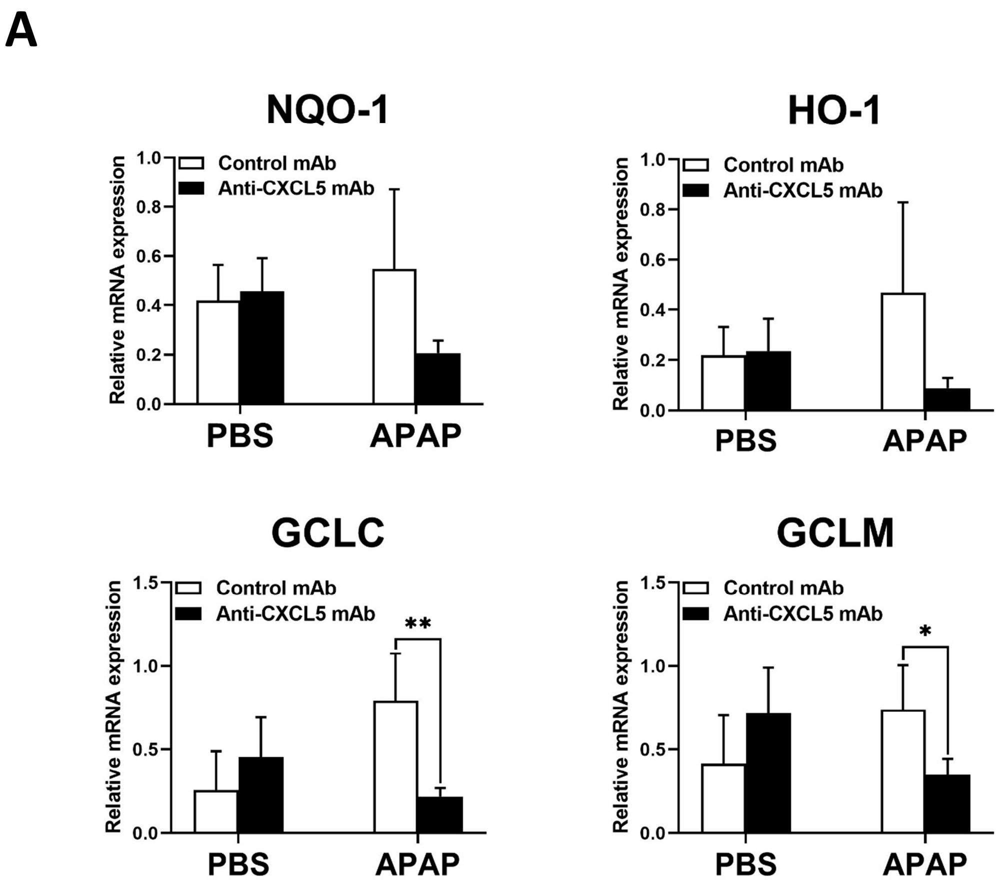

Figure S1. Anti-CXCL5 mAb treatment decreased the mRNA expression levels of Nrf2-target genes in mice with APAP administration. (A) The hepatic mRNA expression levels of NQO-1, HO-1, GCLC, and GCLM were detected. The data are expressed as mean ± SD per group (n = 5 per group). * $p < 0.05$ and ** $p < 0.01$.
